# Supplementary material for: New Insights into the Transcriptional Regulation of Genes Involved in the Nitrogen Use Efficiency under Potassium Chlorate in Rice (Oryza sativa L.)
Source: Int J Mol Sci. 2021 Feb 22;22(4):2192. doi: 10.3390/ijms22042192 (PMC7926690; doi:10.3390/ijms22042192)
Supplement: Supplementary file 1 [file ijms-22-02192-s001.pdf]

New Insights into the Transcriptional Regulation of Genes Involved in the Nitrogen Use Efficiency under Potassium Chlorate in Rice (*Oryza sativa* L.)

Nkulu Rolly Kabange [1], So-Yeon Park [1], Ji-Yun Lee [1], Dongjin Shin [1], So-Myeong Lee [1], Youngho Kwon [1], Jin-Kyung Cha [1], Jun-Hyeon Cho [1], Dang Van Duyen [2], Jong-Min Ko [1] and Jong-Hee Lee [1,*]

[1] Department of Southern Area Crop Science, National Institute of Crop Science, RDA, 50424, Miryang, Korea; N.R.K., rollykabange@korea.kr; S.-Y.P., f55261788@korea.kr; J.-Y.L., minitia@korea.kr; D.-J. S., jacob1223@korea.kr; S.M.L., olivetti90@korea.kr; Y.H.K., kwon6344@korea.kr; J.K.C., jknzz5@korea.kr; J.M.K., kojmin@korea.kr

[2] Molecular Biology Department, Agricultural Genetic Institute, Hanoi, Vietnam; dangvanduyen79@gmail.com

* Correspondence: J.H.L, ccriljh@korea.kr; Tel.: +82-53-350-1168, Fax: +82-55-352-3059

International Journal of Molecular Sciences

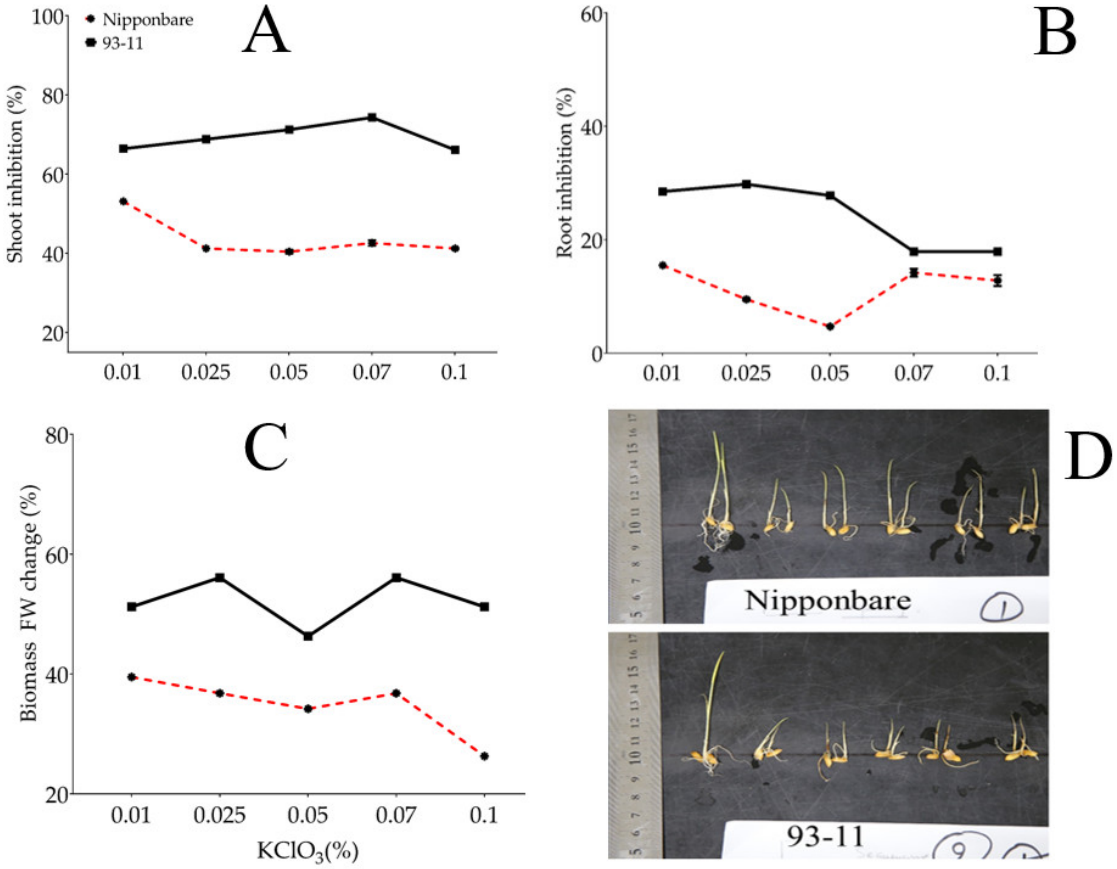

Figure S1. Optimization of potassium chlorate ($KClO_3$) concentration using Nipponbare and 93-11 rice cultivars. (**A**) The pattern of shoot inhibition percentages of Nipponbare (typical *japonica* ssp.) and cv.93-11 (typical *indica* ssp.) in response to gradients $KClO_3$ concentrations (0.01, 0.025, 0.05, 0.07, and 0.1%). (**B**) The roots inhibition pattern under gradient $KClO_3$ concentrations. (**C**) Changes of biomass fresh weight under the same conditions. (**D**) Phenotypes of seedlings exposed to gradients $KClO_3$ concentrations 7 days after treatment.

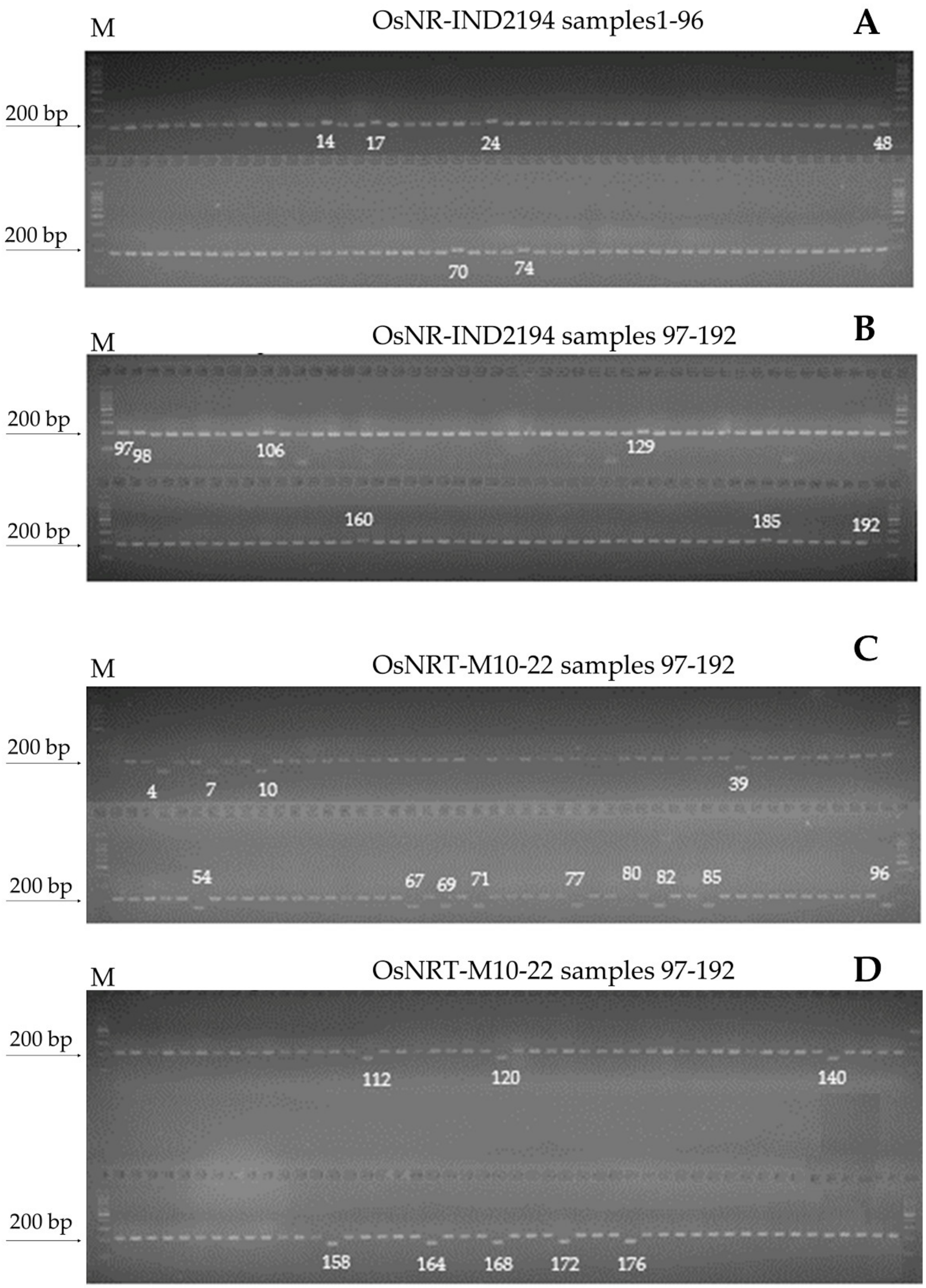

Figure S2. Identified nitrate reductase (NR) and nitrate transporter (NRT) introgression lines. This figure shows the genotyping results of representative rice lines pulled from 420 BC2F7 rice lines derived from a cross between Saeilmi × Milyang23, *japonica* and *indica* subspecies, respectively, using insertion/deletion (InDel) markers. (A–B) NR introgression lines amplifying the *indica* allele of nitrate reductase (200 bp), and (C–D) NRT introgression rice lines amplifying the *indica* allele of nitrate transporter (165 bp).

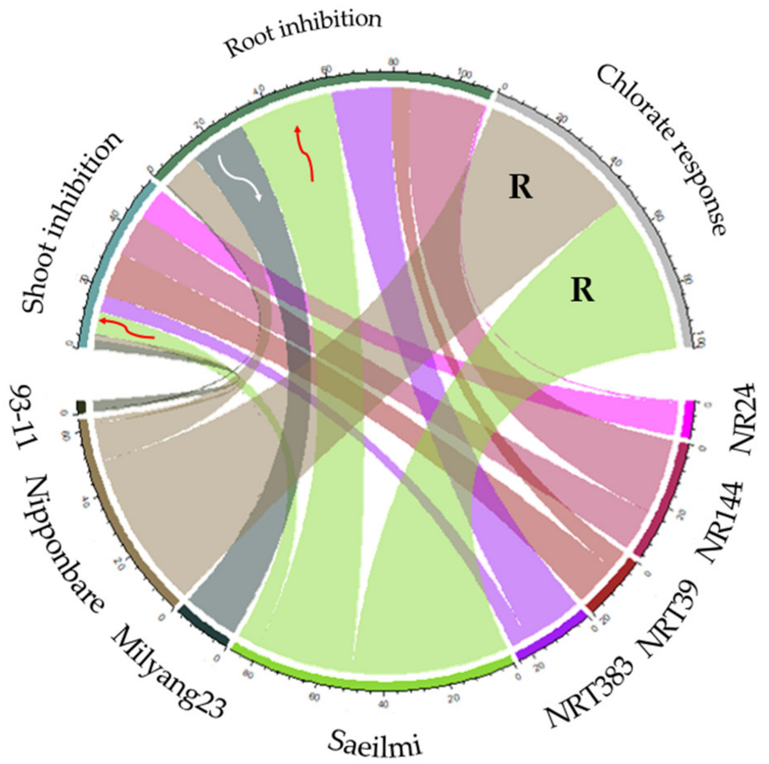

Figure S3. Chord diagram showing the shoot and roots growth patterns of Saeilmi (P1) and Milyang23 (P2), and four BC2F7 introgression lines. This figure shows the proportion of the shoot growth and roots growth, and the potassium chlorate response of two Nitrate reductase (NR) and nitrate transporter (NRT) introgression lines, the parental lines (Saeilmi, P1 *japonica*; Milyang23, P2 *indica*), as well as that of Nipponbare and 93-11, typical *japonica* and *indica* cultivars used as reference for the phenotypic evaluation under $KClO_3$ treatment. The bottom side of the circular Chord diagram comprise the rice lines evaluated, and the upper side is the traits. Bands or links show the linkage between rice lines and traits. The wider the band width the larger the proportion (increase or decrease). The arrow directed downward indicates a reduction in that specific parameter in the parental line, Milyang23. The R indicates resistant. No indication means reduction of that specific trait in the NR or NRT introgression lines under potassium chlorate ($KClO_3$) treatment.

Table S1. List of primer sequences for gene expression used in the study

Marker/gene names	Locus	Forward primer (5′->3′)	Reverse primer (5′->3′)	Length (F/R)	Tm °C (F/R)	GC contents (F/R)	Amplicon size (bp)
Genotyping InDel marker primers							
OsNR-IND2194	LOC_Os02g53130 /BGIOSGA005531	GTGCTGACCTCACGTCCATC	GTAGCCCGAGCTTCTGGTC	20/ 19	59.5/ 59.2	60/63.2	200 (*indica*)/ 188 (*japonica*)
OsNRT-M10-22	LOC_Os10g40600/ BGIOSGA031434	TCGCGTGACAAATATGACAT	CCACTGCAAGATCCAAGTCT	20/ 20	51.3/ 55.4	50/45	165 (*indica*)/ 213 (*japonica*)
High affinity nitrate transporters encoding gene							
OsNRT1.1B	LOC_Os10g40600	GGCTCGACTACTTCTACTGGC	CGAGGCGCTTCTCCTTGTAG	21/20	59.8/59.5	57.1 / 60	102
High affinity ammonium transporters encoding genes							
OsAMT1.3	LOC_Os02g40710	GCGCGCTCTTCTACTACCTC	GTAGTCGTACCCTGTCTGCG	20/ 20	59.5/ 59.5	60/ 60	120
OsAMT2.3	LOC_Os01g61550	CGGATGAACATCAAGGCGTG	TATCCGCCGGAGTAGTCGAT	20/ 20	57.5/ 57.5	55.0/ 55	116
Glutamate synthase involved in the initial step of nitrogen assimilation							
GLU1	LOC_Os01g48960	TGTTGCTGTCAGTTCGCTCT	AAACCCAACAAGGGTGCAGA	20/20	55.4/ 55.4	50.0/ 50.0	140
GLU2	LOC_Os05g48200	AATGCTCTTCCCAACCCTGG	CTGCTGTTAATCCGTGCTGC	20/20	57.5/ 57.5	55.0/ 55.0	110
Nitrate reductase catalyzing the conversion of nitrate (NO_3) to nitrite (NO_2)							
OsNR2	LOC_Os02g53130	TCCTCGCCTACATGCAGAAC	ATGCGCTTGAGCCATTTCAC	20/20	57.5/ 55.4	55.0/ 50.0	112
Nitric oxide biosynthetic genes							
OsNIA1	LOC_Os08g36480	TCGGCAAGCACATCTTCGT	ACTTGGGGTGCTCGTTCTTG	19/ 20	54.9/ 57.5	52.6/ 55.0	138
OsNIA2	LOC_Os08g36500	TGTACCAGGTCATCCAGTCG	CGATGACGTACCACACCTTG	20 /20	57.5/ 57.5	55.0/ 55.0	162
Housekeeping gene							
OsActin1	LOC_Os05g36290	CTAGCGGTCGAACAACTGGT	ACCGGAGGATAGCATGAGGA	20/ 20	57.5/ 57.5	55.0/ 55.0	102
